# ADAM-17 is a poor prognostic indicator for patients with hilar cholangiocarcinoma and is regulated by FoxM1

**DOI:** 10.1186/s12885-018-4294-9

**Published:** 2018-05-18

**Authors:** Xiaodong Jiao, Wenlong Yu, Jianxin Qian, Ying Chen, Peilian Wei, Wenzheng Fang, Guanzhen Yu

**Affiliations:** 1 Department of Oncology, Longhua Hospital Affiliated to Shanghai University of Traditional Chinese Medicine, Shanghai, 200032 China; 2 Department of Surgery, Eastern Hepatobiliary Surgery Hospital, Shanghai, China; 3 Department of Oncology, Eastern Hepatobiliary Surgery Hospital, Shanghai, China; 4 Department of Pathology, Changhai Hospital, Shanghai, China; 5 Department of Oncology, Fuzhou General Hospital, Fuzhou, Fujian Province China; 6 Department of Medical Oncology, Changzheng Hospital, Shanghai, China

**Keywords:** Hilar cholangiocarcinoma, FoxM1, ADAMs, ADAM-17, Prognosis

## Abstract

**Background:**

A-disintegrin and metalloproteinases (ADAMs) are members of a family of multidomain transmembrane and secreted proteins. Specific ADAMs are upregulated in human cancers and correlated with tumor progression and poor outcome, but rarely studied in human hilar cholangiocarcinoma (HC). This study aimed to explore the expression profiles of ADAMs and their potential underlying mechanisms promoting cancer progression.

**Methods:**

mRNA expression of ADAM-9, − 10, − 11, − 12, − 15, − 17, − 28, and − 33 was analyzed in human hilar cholangiocarcinoma (HC) samples. Immunohistochemical (IHC) analysis was used to detect the expression of ADAM-10, − 17, − 28, and FoxM1 in HC. The regulation of ADAM-17 by FoxM1 and their functional study was investigated in vivo and in vitro.

**Results:**

ADAM-10, − 17, and − 28 were upregulated in tumors compared with matched non-cancerous tissues. IHC analysis revealed increased expression of ADAM-10, − 17, and − 28 in HC cells, and ADAM17 seems to be an independent prognostic factor. ADAM-17 is regulated by FoxM1. A decrease in the expression of ADAM-17 by silencing FoxM1 led to an inhibition of cell proliferation, tumor growth, and the production of tumor necrosis factor α. IHC analysis showed co-expression of FoxM1 and ADAM-17 in HC specimens.

**Conclusions:**

The findings of the present study show an important role of the cross-talk among FoxM1, ADAM-17, and TNFa in HC development and progression.

**Electronic supplementary material:**

The online version of this article (10.1186/s12885-018-4294-9) contains supplementary material, which is available to authorized users.

## Background

Hilar cholangiocarcinoma (HC), also known as Klatskin tumor, is a complex and devastating malignancy of the bile duct [1]. Surgical resection is the most effective management for HC and offers a relatively long-term survival when performed during early stages of cancer [2]. Unfortunately, most HC patients are diagnosed at later stages, when the tumor has metastasized to major structures surrounding the bile duct [1, 3]. Even after complete resection, recurrence has been observed in 50%–70% of cases [1]. It has been estimated that the 5-year survival rate after R0 resection of HC ranges from 10% to 40% [1]. In addition, the mechanisms underlying HC development and progression have not been fully elucidated, which in turn limits the use of targeted therapy. Therefore, demonstrating the molecular alterations and regulatory mechanisms involved in the carcinogenesis of HC is imperative.

A-disintegrin and metalloproteinases (ADAMs) participate in various biological functions, including fertilization, cell adhesion and migration, and proteolysis [4, 5]. There is growing evidence that specific ADAMs are dysregulated in human solid cancers [6–8]. These ADAMs regulate the activation of growth factors (e.g., EGF, TGF-a), cytokines (TNF-a), and integrins, which in turn promote tumor growth and metastasis [9]. Moreover, targeting specific ADAMs by selective ADAM inhibitors is considered as a promising therapeutic strategy [10]. In HC, however, the expression profiles of ADAMs and their regulation are not well understood.

Therefore, we aimed to explore the expression levels of specific ADAMs in HC and to determine the clinical roles of upregulated ADAMs in cancers. Furthermore, we identified the most valuable ADAM, as well as analyzed its function and potential regulatory mechanism.

## Methods

### Patient samples and cell culture

Five fresh paired HC and the adjacent non-cancerous tissues were used in the determination of expression levels of specific ADAMs by using RT-PCR. Tissue microarrays of 49 resected HC specimens and 15 matched non-cancerous bile duct tissues were constructed as previously described [11]. All these patients had detailed clinical history and follow-up information (Table 1). The institutional review boards of the Eastern Hepatobiliary Hospital and Changhai Hospital approved this study, and informed consents were obtained from each patient or his or her guardians.

**Table 1:** Association between ADAM-10, ADAM-17, ADAM-28, and FoxM1 expression and clinical variables of hilar cholangiocarcinoma

Variables	N	ADAM-10	*P*	ADAM-17	*P*	ADAM-28	*P*	FoxM1	*P*
Gender									
Male	38	27	0.866	23	0.309	22	0.235	20	0.049
Female	16	11		12		12		13	
Size									
≤ 3 cm	21	12	0.089	9	0.007	10	0.063	9	0.028
>3 cm	33	26		26		24		24	
T stage									
T1–3	7	2	0.009	3	0.192	3	0.238	3	0.288
T4	47	36		32		31		30	
N stage									
N0	18	10	0.092	9	0.107	9	0.163	10	0.554
N1–2	36	28		26		25		23	
Differentiation									
High/moderate	40	30	0.208	27	0.485	24	0.446	22	0.119
low/undifferentiated	14	8		8		10		11	
TNM									
I/II	22	11	0.007	9	0.002	10	0.027	11	0.165
III/IV	32	27		26		24		22	

Human cholangiocarcinoma cell lines (QBC939 and RBE) and HEK293T cell lines were obtained from the Cell Center of the Chinese Academy of Sciences. The conditions for cell culture were as described in a previous report [11].

### Real-time RT-PCR

The relative expression levels of specific ADAMs were detected by real-time RT-PCR by using a SYBR Premix Ex Taq (Perfect real-time) kit (Takara,Japan) in a Rotor Gene 3000 system (Corbet Research, Sydney, Australia). The sequences of the primers for these ADAMs and those of the internal control, GAPDH, are listed in Additional file 1: Table S1.

### Western blot analysis

Western blot analysis was used to detect the protein levels of FoxM1 (K19, Santa Cruz Biotechnology), ADAM-17 (H300, Santa Cruz Biotechnology), and TNFa (10602-RP02, Sino Biological, Inc.) according to standard procedures [12–14].

### Measurement of TNFαrelease

The levels of TNFa were measured in cell-free supernatants of stably transfected FoxM1-overexpressing RBE cells, FoxM1-shRNA QBC939 cells, and the control cells. Quantitation was performed with ELISA detection kits (EH009–96, Excell BIO) following protocols described by the manufacturer [15].

### Immunohistochemical analysis and scoring

**Fig. 1:**
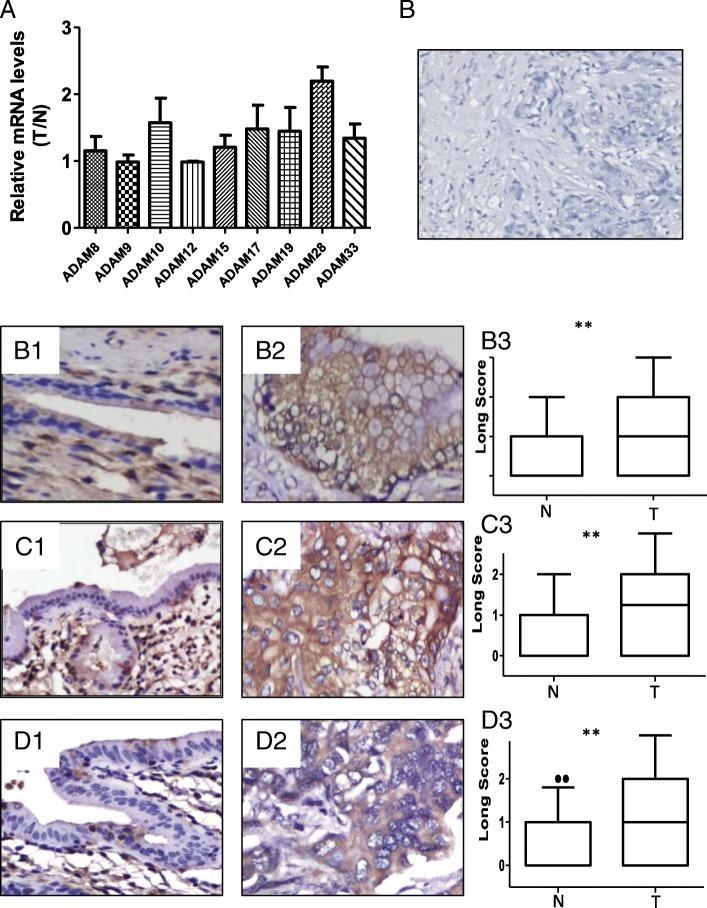
Expression patterns of ADAM proteases in human hilar cholangiocarcinoma and non-cancerous tissues. **a** Normalized mRNA values of specific ADAMs in hilar cholangiocarcinoma tissues compared to non-cancerous tissues. The fold changes (*y axis*) are calculated as the log ratio of the relative amounts of mRNA in tumor/non-cancerous tissues. **b** A negative immunohistochemical control using an isotype Ig antibody. **c, d, e** Expression of ADAM-10 (**c**), ADAM-17 (**d**), and ADAM-28 (**e**) proteins in non-cancerous tissues (C1, D1, and E1) and hilar cholangiocarcinoma (C2, D2, and E2) by immunohistochemical analysis. Magnification: 200×. Graphical representation of the difference in the intensity of ADAM-10 (C3), ADAM-17 (D3), and ADAM-28 (E3) immunostaining in tumor (T) and normal (N) tissues. *P < 0.01

Tissue microarrays containing HC (*n* = 49) and matched non-cancerous bile duct tissues and the fresh tissues (*n* = 5) were immunostained with antibodies against ADAM-10 (1:100), ADAM-17 (1:100), ADAM-28 (1:100, H102, Santa Cruz Biotechnology), and FoxM1 (1:150) according standard protocols. Tissue sections were microwave-treated in citrate buffer (PH 6.0) at 99 °C for 4 min. The sections were placed in 3% H2O2 for 10 min and then washed three times with phosphate-buffered saline (PBS) buffer for 3 min. Normal goat serum was used to block antibody at room temperature for 10 min. The primary antibodies were incubated at 4 °C for 24 h. Biotinylated anti-mouse/rabbit immunoglobulin was used as the second antibody. 3, 3-Diaminobenzidine (DAB) was used as a chromogen. The sections were counter-stained with hematoxylin [16]. An isotype Ig antibody was used as a negative control (Fig. 1b).

Two individuals (G.Y. and Y.C.) independently reviewed the stained sections and scored each case. ADAM expression was scored in epithelial cells (normal or tumor cells). The intensity and percentage of positive tumor cells was determined by using a semi-quantitative scoring system [11]. The mean percentage of positive tumor cells was calculated in five areas of a given sample at a magnification of × 400 and scored from 0 to 1 (0–100%). The intensity of immunostaining was scored as 0 for negative, 1 for weak, 2 for moderate, and 3 for strong. Therefore, a weighted score was generated for each case, ranging from 0 (0% of cells stained) to 300 (100% of cells stained at 3+ intensity) [17]. Finally, the score < 75 was defined as negative/low expression.

### Plasmids and transfections

The pcDNA3.1-FoxM1B and FoxM1-shRNA plasmids were gifts from Prof. Sunyun Huang (MD Anderson Cancer Center, Houston, TX, USA) [18]. QBC cells were transfected with 50 nM of the FoxM1 plasmid or control, whereas RBE cells were infected with 50 nM of shRNA targeting FoxM1 or 50 nM of scrambled siRNA, according to the manufacturer’s instructions. The Fugene-6 system (Qiagen, Crawley, UK) was used for transfection.

### Cell proliferation assay

Approximately 24 h after transfection or infection, the tumor cells were digested and seeded into 96-well plates at a density of 2000 cells per well. CCK8 assays (Dojindo Kumamoto, Japan) were conducted to measure the final absorbance at specific time points.

### Flow cytometric analysis

Flow cytometric analysis was carried out to determine the effects of FoxM1 on cell cycle distribution. Briefly, stable FoxM1-shRNA infected cells and stable FoxM1-overexpression cells were harvested by trypsinization and fixed with 70% ethanol, and measured according to the manufacturer’s protocol (KEY GEN, Nanjing, China). The cell cycle distribution was then analyzed by flow cytometry (FACSCalibur, BD Biosciences, Bedford, MA).

### Subcutaneous tumor growth

Stable FoxM1-shRNA infected cells (density: 1 × 10^7^) were subcutaneously injected into female BALB/c nude mice. Two weeks after injection, the mice were sacrificed, and the tumor was excised, measured, and weighed. Tumor volume (mm^3^) was calculated as V = 0.52 (length × width × depth).

### Statistical analysis

SPSS and GraphPad Prism 5.0 were used in data analysis and for generating representative images. The *t*-test was used to determine the significance of the in vitro results. The χ^2^ test was used to analyze the categorical data. The Kaplan-Meier assay was used to estimate survival rates. The Cox proportional hazards model was used to assess predictors that were related to tumor recurrence and overall survival. *p* < 0.05 was considered as significant [19].

## Results

### Expression profiles of specific ADAMs in HC

Because ADAMs function in the proteolytic processing of other transmembrane proteins, we investigated the expression patterns of proteinase-type ADAM molecules (ADAM8, − 9, − 10, − 12, − 15, − 17, − 19, − 28, and − 33) in HC. Figure 1a shows that the mean mRNA levels of ADAM-10, ADAM-17, ADAM-19, ADAM-28, and ADAM-33 were higher in the tumor samples compared to that in non-cancerous bile duct tissues.

### Relationship between ADAM-10, ADAM-17, and ADAM-28 expression and clinical variables of patients with HC

Considering the importance of ADAM-10, ADAM-17, and ADAM-28 in cancer progression [9], we detected their expression in HC by immunohistochemistry. ADAM-10, ADAM-17, and ADAM-28 were localized to the cytoplasm. The non-cancerous bile ducts showed negative or weak staining for ADAM-10, ADAM-17, and ADAM-28, whereas most cancer cells showed strong staining for these three proteins (Fig. 1b-e). The rate of ADAM-10, ADAM-17, and ADAM-28 high expression in HC was 70.4% (38/54), 64.8% (35/54), and 63.0% (34/54), respectively. Further analysis showed a significant correlation between ADAM-10 overexpression and T stage (*P* = 0.009) and AJCC TNM stage (*P* = 0.007), ADAM-17 and larger tumor size (P = 0.007) and AJCC TNM stage (*P* = 0.002), ADAM-28 and AJCC TNM stage (*P* = 0.027) (Table 1).

**Fig. 2:**
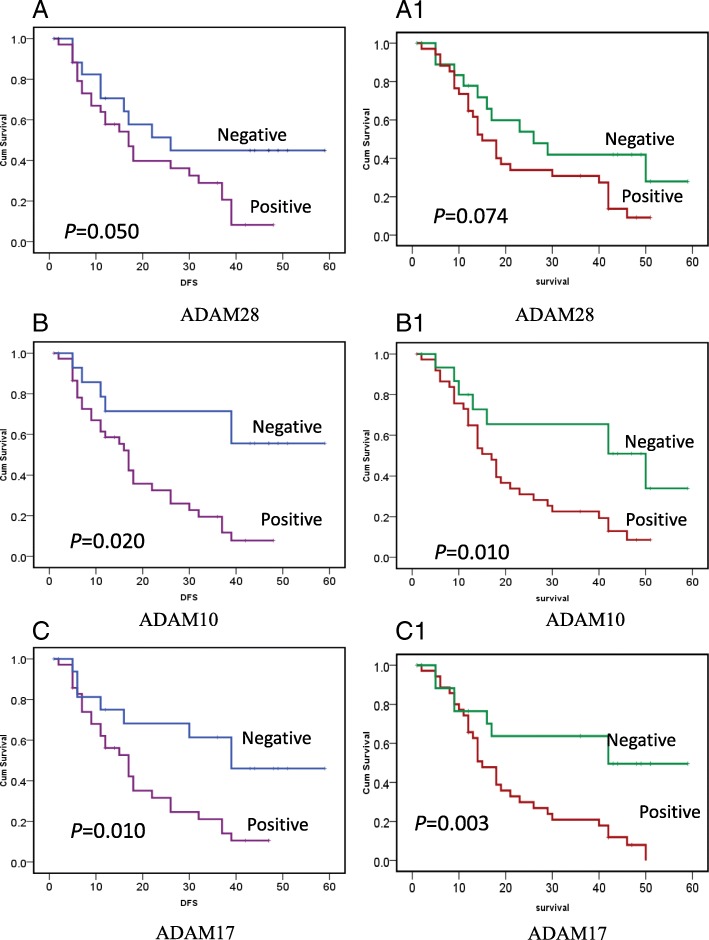
Kaplan-Meier survival patients with hilar cholangiocarcinoma based on ADAM-28, ADAM-10, and ADAM-17 expression. **a, b, c** Cancer overexpressing ADAM-28 (A), ADAM-10 (B), and ADAM-17 (C) showed a higher chance for recurrence than those who did not (ADAM-28: Neg. vs. Pos.: 26mon vs. 15mon, *P* = 0.050; ADAM-10: Neg. vs. Pos.: 41mon vs. 17mon, *P* = 0.020; AD1 M-17: Neg. vs. Pos.: 39mon vs. 17mon, *P* = 0.010). **(A1, B1, C1)** Patients overexpressing ADAM-28 (A1), ADAM-10 (B1), and ADAM-17 (C1) showed a decreased survival duration compared to patients who did not (ADAM-28: Neg. vs. Pos.: 26mon vs. 17mon, *P* = 0.074; ADAM-10: Neg. vs. Pos.: 50mon vs. 17mon, *P* = 0.010; AD1 M-17: Neg. vs. Pos.: 42mon vs. 15mon, *P* = 0.003)

Kaplan-Meier analysis revealed that T stage, regional lymph node metastasis, residual tumor, ADAM-10, ADAM-17, and ADAM-28 were associated with shorter time to tumor progression/recurrence (TTP). All the above covariates were enrolled in the following multivariate analysis. Multivariate analysis showed that the T stage and regional lymph node metastasis were independent factors in predicting tumor recurrence (Fig. 2, Table 2). As for overall survival, T stage, regional lymph node metastasis, residual tumor, ADAM-10, and ADAM-17 were associated with decreased overall survival for patients with HC. Cox proportional hazards model showed that T stage, regional lymph node metastasis, and ADAM-17 were independent prognostic indicators for patients with HC (Fig. 2, Table 3).

**Table 2:** Univariate and Multivariate Analysis of Variables Associated with Recurrence in Patients with hilar cholangiocarcinoma

Variable	No.^a^	TTP (months)	*P*	*P*	HR (multivariate)	95% CI
			(univariate)	(multivariate)		
T stage						
T1–3	7	52	0.002	0.025	0.094	0.012–0.740
T4	46	17				
Regional lymph nodes positive						
No	18	39	0.014	0.021	0.361	0.152–0.858
Yes	35	15				
R						
R0	24	37	0.04	0.926	1.037	0.482–2.232
R1	29	17				
ADAM10						
Negative	15	41	0.002	0.488	0.614	0.155–2.437
Positive	38	17				
ADAM17						
Negative	18	39	0.01	0.485	0.671	0.218–2.059
Positive	35	17				
ADAM28						
Negative	19	26	0.05	0.639	0.813	0.343–1.930
Positive	34	17				

^a^ The detailed information of tumor recurrence of one case was not available

**Table 3:** Univariate and Multivariate Analysis of Variables Associated with Overall Survival (OS) in Patients with hilar cholangiocarcinoma

Variable	No.	OS(months)	*P*	*P*	HR (multivariate)	95% CI
			(univariate)	(multivariate)		
Tumor stage						
T1–3	7	50	0.004	0.02	0.135	0.025–0.730
T4	47	17				
Regional lymph nodes positive						
No	18	42	0.006	0.023	0.384	0.168–0.875
Yes	36	14				
R						
R0	24	26	0.024	0.784	0.903	0.437–1.869
R1–2	30	16				
ADAM10						
Negative	16	50	0.01	0.589	1.359	0.447–4.135
Positive	38	17				
ADAM17						
Negative	19	42	0.003	0.035	0.318	0.109–0.924
Positive	35	15				

### FoxM1B regulates ADAM-17 expression in human HC cell lines

**Fig. 3:**
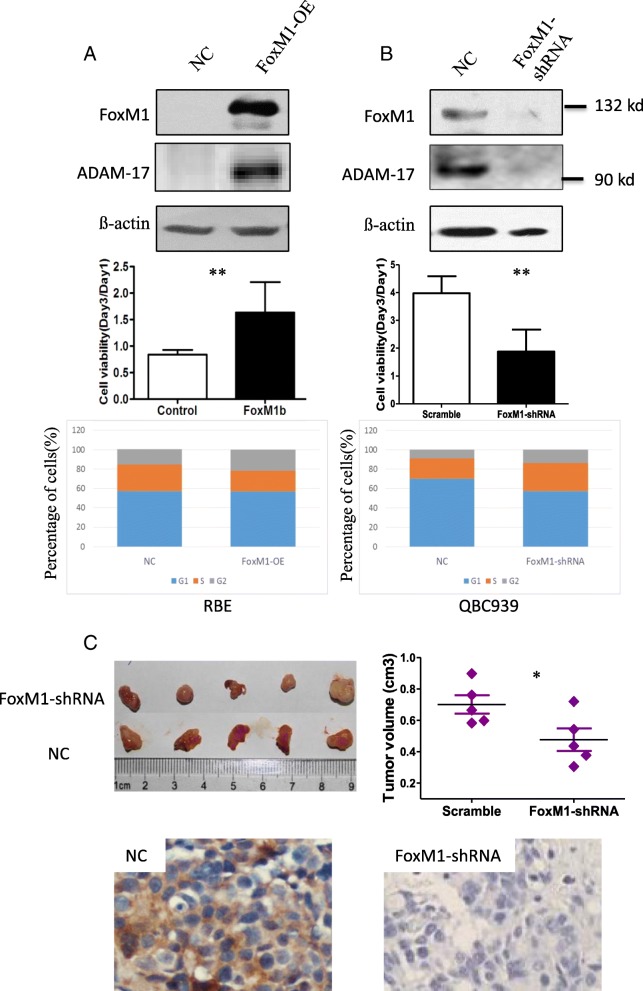
FoxM1 activates ADAM-17 and promotes cell proliferation in vitro and tumor growth in vivo. **a** RBE cells transfected with FoxM1 and control vector after 48 h; FoxM1 and ADAM17 protein expression (upper) were detected by Western blot analysis, and cell viability was analyzed by CCK8 assay (lower). **b** QBC939 cells transfected with FoxM1-shRNA and scrambled control after 48 h; FoxM1 and ADAM-17 protein expression (upper) were detected by Western blot analysis, cell viability was analyzed by CCK8 assay (middle), cell cycle distribution was measured by flow cytometric analysis (lower). All the above experiments were repeated three times. **c** QBC939 cells (1 × 10^7^ per mouse) transfected with FoxM1-shRNA or control shRNA were subcutaneously injected into nude mice (*n* = 5). Resected tumor pictures (left), graphical representation of tumor volumes (right), and staining of ADAM17 in the two groups. OE, overexpression. *p < 0.05, **p < 0.01 as compared to control

Because deletion of FoxM1 induces the downregulation of ADAM-17 [20], we tried to investigate whether ADAM-17 could be regulated by FoxM1 in HC. As expected, overexpression of FoxM1B in RBE cells resulted in an increase in the expression of ADAM-17 and promotion of cell proliferation (Fig. 3a). Silencing of FoxM1 led to a decrease in the expression of ADAM-17, inhibition of cell proliferation and cell cycle arrest, as shown by the accumulation of cells in S phase (Fig. 3b). This might be associated with a change in S phase-related protein cyclin A, which is required to phosphorylate and activate FoxM1 during G(2) phase [21–23]. Next, we examined whether the FoxM1-ADAM-17 axis played a key role on tumor growth by downregulating the expression of FoxM1 and ADAM-17. Fig. 3c shows that tumor volumes were significantly smaller in the FoxM1-knockdown group than that in the control group. Accordingly, IHC staining showed a lower expression of ADAM-17 in FoxM1-knockdown tumors than that in the control tumors.

### Correlation between FoxM1 expression and ADAM-17 expression

**Fig. 4:**
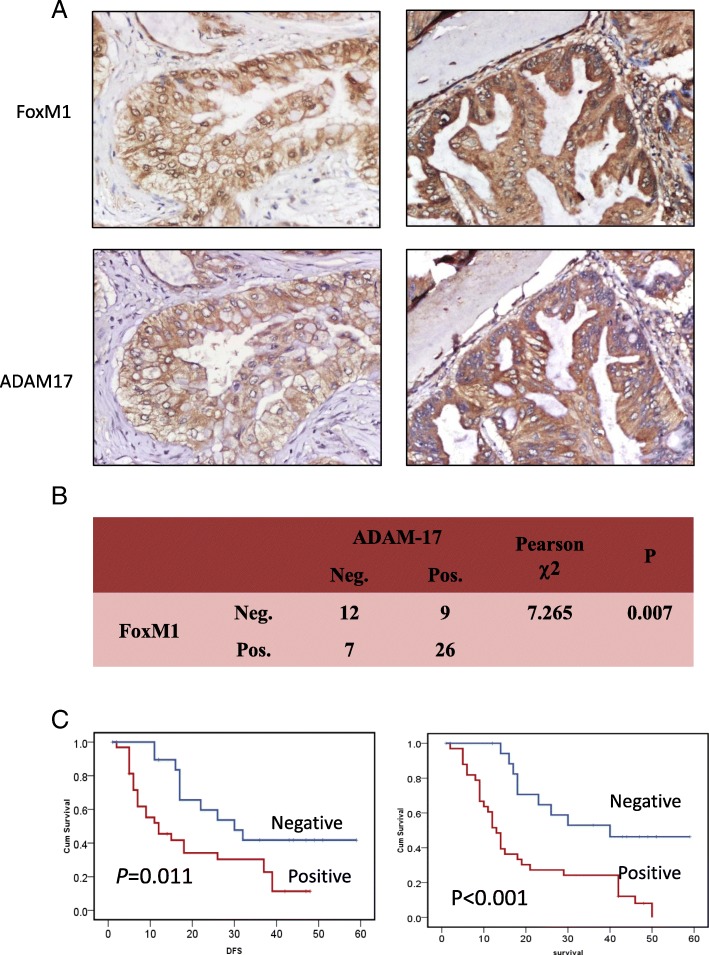
Positive correlations between FoxM1 and ADAM-17 in human hilar cholangiocarcinoma samples. **a** Representative expression of FoxM1 (upper) and ADAM-17 (lower) proteins in tumor samples. Magnification: 40× (left), 200× (right). **b** Kaplan-Meier survival curves of tumor recurrence (left, Neg. vs. Pos.: 30mon vs. 12mon, *P* = 0.011) and overall survival (right, Neg. vs. Pos.: 40mon vs. 13mon, *P* < 0.001) for patients with hilar cholangiocarcinoma based on FoxM1 expression. **c** The correlation between FoxM1 and ADAM-17 expression as determined by Pearson’s correlation test

Next, we detected FoxM1 expression in the 54 cases by immunohistochemistry and analyzed its association with clinical variables and ADAM-17. FoxM1 was overexpressed in 33 of 54 (61.1%) HC cases and was significantly correlated to larger tumor size (*P* = 0.028) (Table 1). IHC staining showed that FoxM1 and ADAM-17 were co-overexpressed in 48.1% (26/54) cases, co-underexpressed in 22.2% (12/54) cases. Only 16 cases of 54 (29.6%) showed un-uniform expression of FoxM1 and ADAM-17(Fig. 4a and b). A significant correlation between FoxM1 expression and ADAM-17 expression was observed (*P* = 0.007) (Fig. 4b). Moreover, patients who showed FoxM1 overexpression had a higher frequency of recurrence than those who did not express this protein (Fig. 4c). Similarly, patients who exhibited FoxM1 overexpression had relatively shorter survival duration than those who did not show FoxM1 expression (Fig. 4c).

### FoxM1 regulates ADAM-17 to promote TNFa production and secretion

**Fig. 5:**
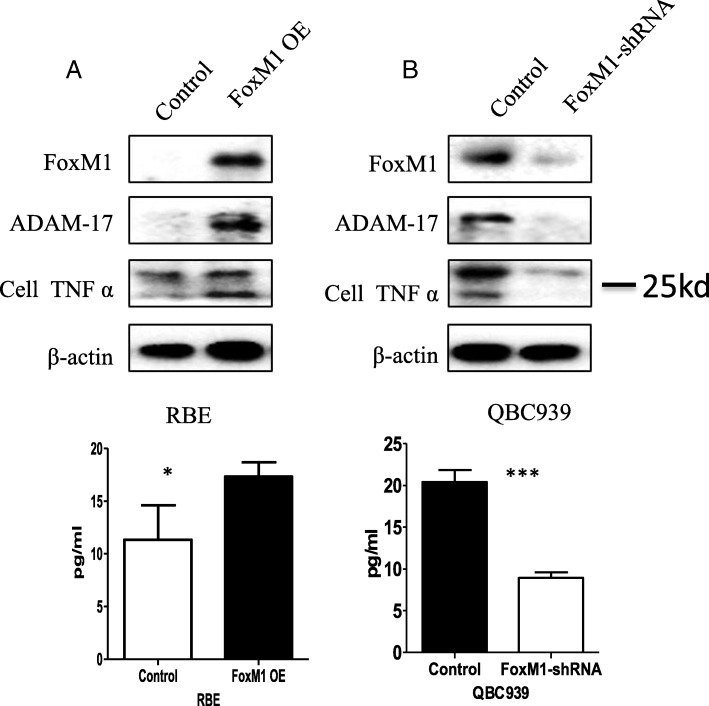
FoxM1 regulates ADAM-17 expression promoting TNFa expression and cleavage. **a** RBE cells were stably transfected with FoxM1 and protein levels of FoxM1, ADAM-17, and TNFa were detected in the whole cell lysates by Western blotting (upper) and TNFa was detected by ELISA in the supernatants (lower). **b** QBC 939 cells were stably transfected with FoxM1-shRNA and protein levels of FoxM1, ADAM-17, and TNFa were detected in the whole cell lysates by Western blotting (upper) and TNFa was detected by ELISA in the supernatants (lower). OE: overexpression. *P < 0.05; ***P < 0.001

In cell adhesion, TNFa undergoes proteolytic processing by ADAM-17. In the present study, we detected the protein expression of TNFa in tumor cell lysates and cell supernatants after FoxM1 was overexpressed or silenced. Undoubtedly, the overexpression of FoxM1 increased the expression of ADAM-17, as well as TNFa expression and cleavage (Fig. 5a). Furthermore, silencing of FoxM1 decreased the expression of ADAM-17, as well as the expression and secretion of TNFa (Fig. 5b).

## Discussion

In the present study, we systemically analyzed the expression of certain proteinase-type ADAMs mRNA in HC and explored the clinical values of ADAM-10, ADAM-17, and ADAM28 in HC. ADAM-17 was regulated by FoxM1. FoxM1 was significantly correlated to ADAM-17 overexpression. The upregulation of FoxM1 and ADAM-17 led to an increase in the activities of cell proliferation and tumor growth, as well as TNFa expression and cleavage, whereas the downregulation of FoxM1 and ADAM-17 showed the opposite results. These findings thus indicate that ADAM-17 may be utilized as an independent prognostic factor in patients with HC. The results also illustrate the critical role of FoxM1/ADAM-17/TNFa in the development and progression of HC.

ADAMs possess an a-disintegrin and metalloprotease domain, indicating its potential role in the progression of human cancers via cell adhesion and protease activities. Consistent with this implication, increasing evidences have suggested that specific ADAMs, including ADAM-9, ADAM-12, ADAM-17, ADAM-28, ADAM-33, were upregulated in various human solid cancers and correlated to the malignant behaviors of these tumors [24–29]. Certain ADAMs may also be utilized as predictors of patients’ outcomes. However, the expression or regulation of ADAMs in HC was not comprehensively examined. Here, we gave the first evidences that certain ADAMs such as ADAM-10, ADAM-17, ADAM-19, ADAM-28, and ADAM-33, were evaluated in HC. Among these, ADAM-10, ADAM-17, and ADAM-28 were the mostly well-studied molecules. We then analyzed their clinical indications in HC. The protein expression levels of ADAM-10, ADAM-17, and ADAM-28 were upregulated in the HC tissues and correlated significantly with disease progression. Interestingly, patients showing ADAM overexpression showed shorter times to recurrence and a poor outcome. These findings suggest that ADAMs are involved in tumor formation and progression of HC.

ADAM activities can be activated by TGF-ß, TPA, PKCδ, and MMPs and inactivated by TIMPs [9]. Considering the importance of ADAM-17 in tumor progression and predicting patient outcomes, we explored the underlying mechanisms of ADAM-17 upregulation. Previous reports have shown that ADAM-17 mRNA levels were remarkably reduced in the lungs of knockout FoxM1 mice [20]. Therefore, it is assumed that FoxM1, a transcription factor, could regulate the expression of ADAM-17. In the present study, we confirmed that FoxM1 regulated ADAM-17 expression in vivo and in vitro, leading to enhanced cell proliferation and tumor growth. Further investigation indicated that FoxM1 is co-expressed with ADAM-17 in the majority of HC samples. These findings offer evidence that FoxM1 regulates ADAM-17 expression, which may significantly contribute to the invasiveness and malignant potential of cancer cells.

ADAM-17, known as TNFa-converting enzyme (TACE), can activate the tissue inflammatory cytokine, Pro-tumor Necrosis Factor a (Pro-TNFa), to biologically active, free TNFa [30, 31]. In the present study, the enhanced expression of ADAM-17 by FoxM1 increased TNFa production and cleavage, whereas the decreased expression of ADAM-17 by FoxM1-shRNA resulted in a decrease in TNFa production and cleavage. Activated TNFa in turn releases various cytokines (such as interleukins), which promotes cell viability and cancer progression [32]. This result further confirmed that FoxM1 is a key regulator of the ADAM-17/TNFa axis in cancer cell proliferation and progression.

## Conclusions

In summary, we showed that specific ADAMs are overexpressed in HC and correlated with tumor invasion and disease progression. ADAM-17 is an independent prognostic marker for patients with HC. FoxM1 regulates the expression of ADAM-17 and then induced TNFa production and cleavage. Therefore, the FoxM1/ADAM-17/TNFa axis might be a promising targeted pathway for the development of therapeutic strategies of HC.

## Additional file

Additional file 1: **Table S1.** Primers of selected genes involved in this study. The detailed information of the primers of selected ADAMs in this study. (DOCX 14 kb)

## Abbreviations

ADAMs: A-disintegrin and metalloproteinases
HC: Hilar cholangiocarcinoma
IHC: Immunohistochemical
