# Supplementary material for: ADAM-17 is a poor prognostic indicator for patients with hilar cholangiocarcinoma and is regulated by FoxM1
Source: BMC Cancer. 2018 May 18;18:570. doi: 10.1186/s12885-018-4294-9 (PMC5960197; doi:10.1186/s12885-018-4294-9)
Supplement: Supplementary file 1 — Table S1. Primers of selected genes involved in this study. The detailed information of the primers of selected ADAMs in this study. (DOCX 14 kb) [file 12885_2018_4294_MOESM1_ESM.docx]

| **Gene Symbol** | **Forward primer** | **Reverse primer** | **Product length** |
| --- | --- | --- | --- |
| ADAM8 | GTTCCAGCATTCCACGG | GGAGCCTCTCAGGTAGAT | 105 |
| ADAM9 | ATACTGCATTGAGGGACG | AGTAGCTTCTCCACAGTTG | 108 |
| ADAM10 | GAGAAGTGTCGGGATGATT | TACAGTCTGTGAAGTTTGGTT | 103 |
| ADAM12 | AGAAGTGTGGGAACAGATTT | GGCTTCAGGGTACAGGT | 106 |
| ADAM15 | GTGTGAACATGGACCACT | AGCTATTCCCAGGCAAATC | 105 |
| ADAM17 | TGTCCTACTGCACAGGTAAT | GTTCCCTCTCGCAGAAAG | 123 |
| ADAM19 | GTATGGCTGCATGTGACAA | CTGTCTACACTGCCTACGA | 117 |
| ADAM28 | GTGTGCAGACCAGCAAA | GTGATGGCAAGGGAAGC | 108 |
| ADAM33 | GGAGATGCTCATGGAAACT | GCCATCTAGGTGAACGGTA | 159 |
| hsa GAPDH | TGTTGCCATCAATGACCCCTT | CTCCACGACGTACTCAGCG | 201 |

Table S1. Primers of selected genes involved in this study
